# *Notes from the Field:* Outbreak of Multidrug-Resistant *Shigella sonnei* Infections in a Retirement Community — Vermont, October–November 2018

**DOI:** 10.15585/mmwr.mm6817a5

**Published:** 2019-05-03

**Authors:** Jonathan Strysko, Veronica Fialkowski, Zachary Marsh, Ashutosh Wadhwa, Jennifer Collins, Radhika Gharpure, Patsy Kelso, Cindy R. Friedman, Kathleen E. Fullerton

**Affiliations:** ^1^Epidemic Intelligence Service, CDC; ^2^Division of Foodborne, Waterborne, and Environmental Diseases, National Center for Emerging Zoonotic and Infectious Diseases, CDC; ^3^Vermont Department of Health; ^4^Laboratory Leadership Service, Division of Scientific Education and Professional Development, CDC; ^5^Division of Healthcare Quality and Promotion, National Center for Emerging Zoonotic and Infectious Diseases, CDC.

On October 22, 2018, the Vermont Department of Health (VDH) notified CDC’s Waterborne Disease Prevention Branch of an outbreak of diarrhea caused by *Shigella sonnei* among residents, visitors, and staff members of a retirement community in Chittenden County, the state’s most populous county. High-quality single nucleotide polymorphism (SNP) analysis predicted initial isolates were multidrug resistant (MDR), and were closely related to a concurrent multistate cluster (differing by 0–11 SNPs). In the United States, rates of MDR shigellosis are increasing (*1*); outbreaks of MDR shigellosis are more common among men who have sex with men and are rare in retirement community settings (*2*). CDC collaborated with VDH to identify additional cases, determine transmission routes, and recommend prevention and control measures.

A confirmed case was defined as isolation of *S. sonnei* from the stool of a facility resident, visitor, or staff member during October 1–November 8. A probable case was defined as diarrheal illness without a positive culture in this population during the same period. Overall, 75 cases (24 confirmed and 51 probable) with onset dates from October 9 through November 3 were identified (Figure), including six cases in visitors to the facility. The attack rate was 15% (46 of 311) among residents and 11% (23 of 209) among staff members. The median patient age was 80 years (range = 21–99 years); 75% were female. Six patients were hospitalized (median duration of hospitalization = 4 days; range = 2–10 days). Two patients, both of whom had other serious comorbidities, died; shigellosis was not thought to be the primary cause of death in these patients. Antibiotic susceptibility testing at CDC determined that outbreak isolates were resistant to trimethoprim-sulfamethoxazole, ampicillin, and ceftriaxone and had decreased susceptibility to azithromycin.

**FIGURE:**
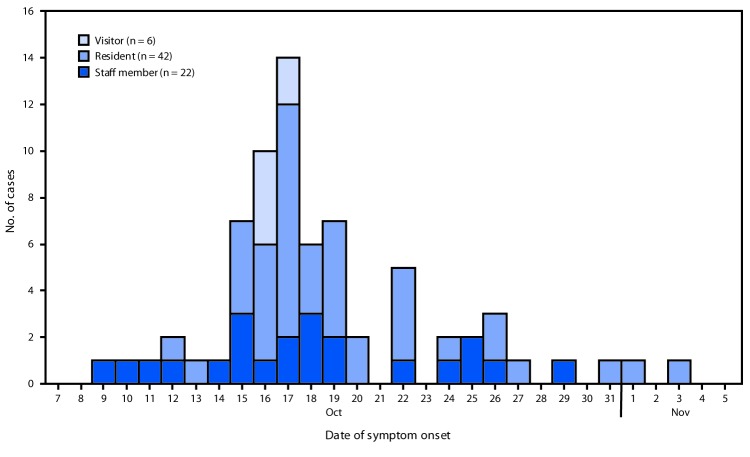
Confirmed and probable cases of shigellosis at a retirement community outbreak, by date of illness onset and facility affiliation (N = 70*) — Vermont, October–November, 2018 * Five patients (four residents and one staff member) had illness onset within the outbreak period of October 1–November 8 but are not included in figure because exact illness onset date was not known.

A review of facility records and key informant interviews identified early cases among one staff member who prepared food while ill during October 11–14 and among six visitors who dined at the facility on October 14th. This information supported foodborne transmission as a leading hypothesis for spread within the facility. A case-control study was conducted using a standardized questionnaire administered to residents and staff members asking about meal exposures and other known risk factors for shigellosis. Controls were residents and staff members at the facility during October 1–November 8 who met neither the probable nor confirmed case definitions. Thirty-six case-patients and 172 controls were included in the analysis. Illness was associated with eating several facility meals during October 11–14, with the strongest associations being dining at the facility on October 14 (odds ratio [OR] = 5.6; 95% confidence interval [CI] = 2.4–14.1), specifically at brunch (OR = 5.5; 95% CI = 2.3–13.3) and breakfast (OR = 5.3; 95% CI = 1.2–22.9). Illness was not associated with attending large gatherings, and no patient reported recent sexual contact or recreational water use. Patient interviews did not identify a direct epidemiologic link with the concurrent multistate cluster.

Food handling was an important mode of transmission of shigellosis within this facility. Reports of staff members working while ill highlights the importance of having clear, nonpunitive sick leave policies. This outbreak investigation also demonstrates that MDR shigellosis can affect a range of populations and underscores the need for evidence-based prevention strategies for all vulnerable groups.
